# Effects of intraoperative blood loss during liver resection on patients’ outcome: a single-center experience

**DOI:** 10.3906/sag-2008-78

**Published:** 2021-06-28

**Authors:** Muhammed Selim BODUR, Kadir TOMAS, Serdar TOPALOĞLU, Şükrü OĞUZ, Hakan KÜÇÜKASLAN, Davut DOHMAN, Erdem KARABULUT, Adnan ÇALIK

**Affiliations:** 1 Department of Surgery, School of Medicine, Karadeniz Technical University, Trabzon Turkey; 2 Department of Radiology, School of Medicine, Karadeniz Technical University, Trabzon Turkey; 3 Department of Anesthesiology, School of Medicine, Karadeniz Technical University, Trabzon Turkey; 4 Department of Biostatistics, Hacettepe University, Ankara Turkey

**Keywords:** Liver surgery, operative bleeding, portal triad clamping, low central venous pressure, hemostasis

## Abstract

**Background/aim:**

Operative bleeding is one of the major determinants of outcome in liver surgery. This study aimed to describe the impact of intraoperative blood loss on the postoperative course of liver resection (LR).

**Materials and methods:**

The data of 257 patients who were treated with LR between January 2007 and October 2018 were retrospectively analyzed. LRs were performed via intermittent portal triad clamping (PTC) under low central venous pressure.

**Results:**

LRs were performed for 67.7% of patients with a malignant disease and 32.3% of patients with a benign disease. Major LR was performed in 89 patients (34.6%). The mean PTC period was 20.32 min (±13.7). The median intraoperative bleeding amount was 200 mL (5–3500 mL), the 30-day mortality rate was 4.3%, and the morbidity rate was 31.9%. The hospital stay (p = 0.002), morbidity (p = 0.009), and 30-day mortality (p = 0.041) of patients with a bleeding amount of more than 500 mL significantly increased.

**Conclusion:**

Surgeons should consider the adverse effects of intraoperative bleeding during liver resection on patients’ outcome. Favorable outcomes would be obtained with diligent postoperative care.

## 1. Introduction

The need of perioperative blood transfusion is reported between 20% and 50% of major LRs [1–5]. Operative blood loss and exposure to allogenic blood are associated with poor outcomes in patients undergoing LR [1–3]. 

Liver-specific complications seen after LR are biliary leak, bleeding, and posthepatectomy liver failure (PHLF). In the early studies, bleeding was the primary cause of overall mortality following LR. The mortality for hepatic resections has improved significantly since the 90s and the bleeding accounts for a minor percentage of overall morbidity in the current series. However, other outcomes have not improved in parallel with mortality such that overall morbidity is still reported in the range of 14% to 45% [4]. 

Some of the important strategies to control blood loss during LR are afferent or complete devascularization before parenchymal transection, low central venous pressure (CVP) surgery, and temporary occlusion of blood inflow with or without outflow control [6]. For precise hemostasis during parenchymal transection, devices such as ultrasonic dissectors, heat coagulants, or bipolar vessel sealants are used [7,8].

In this study, we presented the results of our policy on controlling bleeding and hemostasis during LR. We also briefly discussed the strategies to minimize blood loss in liver surgery.

## 2. Materials and methods

A total of 271 patients underwent LR between January 2007 and November 2018 in Karadeniz Technical University Hospital. Exclusion criteria were as follows: age of 16 years or younger, surgery on the liver without parenchymal resection (including inoperable cases), and LR performed because of liver trauma (n = 5). The authors analyzed only the data of the first LR attempts in patients who underwent liver reresection (n = 16). Overall, 257 patients were considered eligible for this study. The patients’ data were prospectively recorded and retrospectively analyzed. 

### 2.1. Patient selection

The status of the functional liver was evaluated in terms of Child–Pugh–Turcotte (CPT) scores. Patients with a CPT score greater than seven and a model for end-stage liver disease score greater than 16 were considered for the liver transplantation. Future liver remnants were calculated with the help of volumetric analyses from CT images. In the presence of biliary obstruction upon admission, cholestasis was managed with percutaneous transhepatic biliary drainage before the operation. The patients were monitored without operation until the total bilirubin level decreased to 3 mg/dL. 

### 2.2. Liver resection and perioperative care

CVP was maintained at less than 5 cm H2O during LR. LRs were performed through a J-shaped right subcostal incision. A conventional technique including intermittent PTC in 15/5 min cycles of clamp/unclamp times has been used for LRs [2,9–11]. The liver was transected via clamp crushing and a vessel sealing device (LigaSureTM, Medtronic plc, Minneapolis, MN, USA). Major LR was defined as the resection of three or more segments. A variety of LR types are listed according to diagnosis in Table 1. After macroscopic leakage was initially controlled, a fibrin sealant (Tisseel, Baxter Healthcare Corp., Westlake Village, CA, USA) was applied to the cut surface. The whole part of the cut surface was then coated with oxidized cellulose (Surgicel Fibrillar, Ethicon SÀRL, Puits-Godet 20, Neuchâtel, Switzerland). Resection of common hepatic duct was required in patients with Klatskin tumor (n = 22/22) and alveolar hydatid disease (n = 3/16) related to disease invasion or disease extension (in a patient with hepatholithiasis). Hepaticojejunostomy was carried out with interrupted 5/0 polydioxanone (PDS) sutures. The operation field was drained with classic perforated Jackson Pratt silicone drains (Cardinal Health, Dublin, OH, USA). The fascial planes of the incision were closed with a running no. 1 PDS suture [10]. Antibiotic prophylaxis composed of ceftriaxone (1 × 2 g/day) was initially given within 1 h before incision and continuously administered for 5 postoperative days (PODs) to patients without systemic inflammatory response syndrome. In the presence of bilioenteric anastomosis, metronidazole was added to the ceftriaxone treatment. The patients also received prophylactic daily subcutaneous injection of low-molecular-weight heparin (LMWH) beginning POD 0. LMWH treatment was continued until the first postoperative month was completed.

**Table 1:** Types of liver resection.

Malignant diseases, n (%)						Benign diseases, n (%)					
Diagnosis	Major LR,62 (35.6)		Minor LR,112 (64.4)		N = 174	Diagnosis	Major LR,27 (32.5)		Minor LR, 56 (67.5)		N = 83
HCC	RH	6	Sec.	16	69	Giant hemangioma	RH	1	Sec.	9	44
	ERH	3	Seg.	29			LH	2	Seg.	23	
	LH	4	NASSLR	6			ELH	1	NASSLR	3	
	ELH	2					Seg.≥3	5			
	Seg.≥3	3				Hydatid cystof the liver			Sec.	5	8
Klatskin tumor	RH	4			22				Seg.	3	
	ERH	3				Alveolarhydatiddisease	RH	3	Sec.	2	16
	LH	10					ERH	1	Seg.	3	
	ELH	5					LH	2			
IHCC	RH	2	Seg.	2	8		ELH	2			
	ELH	1	NASSLR	2			Seg.≥3	3			
	Seg.≥3	1				Hepatolithiasis	RH	1	Sec.	1	7
Gallbladder carcinoma	RH	1	Seg.	7	10		LH	5			
			NASSLR	2		Granuloma			NASSLR	5	5
CRC metastasis	RH	3	Sec.	14	52	Focal nodular hyperplasia	RH	1	Seg.	2	3
	ERH	2	Seg.	9							
	ELH	1	NASSLR	14							
	Seg.≥3	9									
Breast carcinoma metastasis	Seg.≥3	1	Seg.	2	4						
			NASSLR	1							
NET metastasis			Sec+NASSLR	1	1						
Lymphoma	RH	1	Seg.	1	2						
Gastric cancer metastasis			Seg.	1	1						
Testis tumor metastasis			NASSLR	1	1						
Bladder carcinoma metastasis			Seg.	1	1						
RCC metastasis			Seg.	1	1						
Parotid tumor metastasis			Seg.	1	1						
GIST metastasis			Seg.	1	1						

Abbreviations: LR, liver resection; RH, right hepatectomy; ERH, extended right hepatectomy; LH, left hepatectomy, ELH, extended left hepatectomy; Seg., segmentectomy; Sec., sectorectomy; NASSLR, nonanatomical subsegmentary liver resection; HCC, hepatocellular carcinoma; IHCC, intrahepatic cholangiocarcinoma; CRC, colorectal carcinoma; NET, neuroendocrine tumor; RCC, renal cell carcinoma; GIST, gastrointestinal stromal tumor.

### 2.3. Postoperative follow-up and data collection

The patients were provided with standardized pulmonary care 1 day after extubation [2]. Drains were removed within 5 PODs in the absence of a suspicious content of hemorrhage or biliary leak. PHLF was diagnosed in accordance with the International Study Group of Liver Surgery (ISGLS) criteria [12]. In the case of PHLF, our algorithm was applied to support the failing liver [13]. 

### 2.4. Statistical analysis

Receiver operating characteristic (ROC) curves were used to determine the optimal cut point of the total bleeding volume. The maximum value of Youden index was used to divide the patients into two groups in terms of operative bleeding (for 500 mL, sensitivity = 0.284 and specificity = 0.898). Categorical variables were comparatively analyzed using a chi-square test and Fisher’s exact test. Quantitative parameters were examined using an independent sample t-test or the Mann–Whitney U test as appropriate. Univariate and multivariate analyses were performed using the Cox proportional hazard regression model. Differences at p < 0.05 were considered statistically significant.

## 3. Results

The demographic and operative parameters were summarized in Table 2. Overall morbidity rate was 31.9%. Pulmonary complications were the leading causes of morbidity (n = 70/82). Pneumonia was evolved to sepsis in four patients. Pulmonary embolism (PE) was the worst complication (mortality rate of 60%) in the study group. The incidence of biliary leak was 6.6% (n = 17). The biliary leak originated from the cut surface of the remnant liver (n = 11), the bilioenteric anastomosis (n = 5), or the stump of the right hepatic duct (n = 1). Biliary leak was controlled with relaparotomy (n = 3) or percutaneous drainage (n = 10). Surgical site infections (SSIs) were observed in 15 cases (5.8%). Eight patients suffered from superficial SSIs, whereas four patients had deep SSI. Three patients with organ/space SSIs had dehiscence. Furthermore, 14 (5.4%) cases had PHLF that generally developed after major LR (n=11/14) and resection for malignancy (n=11/14). Six of the 14 cases with PHLF had chronic liver disease before operation. Five patients were in stage C, six patients were in stage B, and three patients were in stage A. With the help of our treatment policy for PHLF, 11 of the 14 patients survived. Seven patients were reoperated within the early postoperative period (30-day after operation) because of biliary leak (n = 2), biliary obstruction (n = 1), dehiscence secondary to organ/space SSIs (n = 3), and postoperative bleeding (n = 1). The multivariate analyses indicated that malignant etiology and transfusion of erythrocyte suspension were independent risk factors for the development of morbidity after liver resection (Table 3). 

**Table 2:** Univariate analysis of risk factors for morbidity and hospital mortality.

Variable	n	Morbidity	p	Mortality	p
SexMale Female	149108	51 (34.2%)31 (28.7%)	0.348	7 (4.7%)5 (4.6%)	0.611
Age (years, mean ± SEM)	56.33 (±13.61)	59.34 (±12.71)	0.015	61 (±10.91)	0.246
BMI (kg/m2, Mean ± SEM)	26.41 (±4.41)	26.68 (±4.29)	0.487	25.64 (±3.99)	0.633
ASAASA 1ASA 2ASA 3ASA 4	80141342	16 (20%)51 (36.2%)13 (38.2%)2 (100%)	0.008	4 (5%)3 (3.5%)3 (8.8%)0 (0%)	0.403
DMYesNo	33224	17 (51.5%)65 (29%)	0.010	2 (6.1%)10 (4.5%)	0.656
COPDYesNo	14243	5 (35.7%)77 (31.7%)	0.479	1 (7.1%)11 (4.5%)	0.497
CADYesNo	16241	5 (31.3%)77 (32.1%)	0.945	2 (12.5%)10 (4.2%)	0.167
HBV-positiveYesNo	48209	14 (29.2%)68 (32.5%)	0.652	2 (4.2%)10 (4.8%)	1.000
HCV-positiveYesNo	19238	10 (52.6%)72 (30.3%)	0.044	1 (5.3%)11 (4.6%)	1.000
DiagnosisBenignMalignant	83174	15 (18.1%)44 (38.5%)	0.001	0 (0%)12 (6.9%)	0.011
Cirrhotic liverYesNo	57200	23 (40.4%)59 (29.5%)	0.121	3 (5.3%)9 (4.5%)	0.732
Extent of resectionMinorMajor	16889	44 (26.2%)38 (42.7%)	0.004	5 (2.9%)7 (7.9%)	0.113
Extrahepatic procedureYesNo	57200	25 (43.9%)57 (25.8%)	0.028	5 (8.8%)7 (3.5%)	0.146
Operation time (min, mean ± SEM)	155.74 (±86.62)	184.71 (±105.67)	0.014	205.42 (±135.64)	0.358
PTC period (min, mean ± SEM)	20.32 (±13.7)	21.09 (±14.05)	0.400	16.75 (11.78%)	0.567
Blood loss <500 mL≥500 mL	18968	53 (28.0%)29 (42.6%)	0.027	6 (3.2%)6 (8.8%)	0.088
ES transfusionYesNo	72185	37 (51.4%)45 (24.3%)	<0.001	8 (11.1%)4 (2.2%)	0.005
FFP transfusionYesNo	18968	66 (34.9%)16 (23.5%)	0.084	10 (5.3%)2 (2.9%)	0.738

Abbreviations: ASA, American Society of Anesthesia; BMI, body mass index; CAD, coronary artery disease; COPD, chronic obstructive pulmonary disease; DM, diabetes mellitus; ES, erythrocyte suspension; HBV, hepatitis B virus; HCV, hepatitis C virus; PTC, portal triad clamping; FFP, fresh frozen plasma; SEM, standard error of the mean.

**Table 3:** Significant factors for morbidity by mutivariant analysis.

	Univariate		Multivariate	
Variable	OR (95% CI)	p	OR (95% Cl)	p
Age	1.03 (1.01–1.05)	0.016		
ASAASA 2ASA 3–4	2.27 (1.19–4.33)2.86 (1.21–6.75)	0.0210.0130.017		
DM	2.60 (1.24–5.45)	0.012		
Diagnosis (benign/malignant)	2.84 (1.50-5.39)	0.001	2.74 (1.43–5.28)	0.003
Extent of resection	2.22 (1.29–3.83)	0.004		
Extrahepatic procedure	1.96 (1.07–3.60)	0.03		
Blood loss	1.91 (1.07–3.39)	0.028		
ES transfusion	3.29 (1.86–5.82)	<0.001	3.20 (1.78–5.73)	<0.001
FFP transfusion	1.74 (0.92–3.29)	0.086		

The Cox proportional hazard regression model was used. Abbreviations: CI, confidence interval; OR, odds ratio.

Overall, the 30-day mortality rate was 4.3%. The patients died from PE (n = 3), sepsis (n = 4), PHLF (n = 3), and acute myocardial infarction (MI, n = 1). The progression of PHLF was not controlled in three patients who died within the first postoperative week. All the patients who died from PHLF had chronic liver diseases (Hepatitis B virus infection, n = 2 and Hepatitis C virus infection, n = 1) and two of them underwent major LR. 

The morbidity rate, hospital stay, and the mortality rate of the patients with a bleeding amount of more than 500 mL significantly increased compared to that of the patients with a bleeding amount of less than 500 mL (Table 4). 

**Table 4:** The comparison of variables regarding bleeding amount in operation.

Parameters	Bleeding amount <500 mL(n = 189)	Bleeding amount ≥500 mL(n = 68)	p-value
Age, mean (±SEM)	55.79 (±13.65)	57.85 (±13.51)	0.284
Sex, n (%)MaleFemale	102 (53.9)87 (46.1)	47 (69.1)21 (30.9)	0.032
Liver lesion, n (%)Benign Malignant	65 (34.3)124 (65.7)	18 (26.4)50 (73.6)	0.290
ASA score, n (%)ASA 1ASA 2ASA 3ASA 4	66 (34.9)95 (50.2)27 (14.4)1 (0.5)	14 (20.5)46 (67.6)7 (10.5)1 (1.4)	0.207
BMI, kg/m2, mean (±SEM)	26.53 (±4.27)	26.08 (±4.78)	0.474
DM, n (%)	24 (12.6)	9 (13.2)	1.000
COPD, n (%)	12 (6.3)	2 (2.9)	0.366
CAD, n (%)	12 (6.3)	4 (5.8)	1.000
HBV infection, n (%)	33 (17.4)	15 (22)	0.468
HCV infection, n (%)	16 (8.4)	3 (4.4)	0.418
Presence of cirrhosis	38 (20.1)	19 (27.9)	0.233
Liver resection, n (%)MinorMajor	142 (75.1)47 (24.9)	26 (38.2) 42 (61.8)	<0.001
Additional surgery, n (%)	38 (20.1)	19 (27.9)	0.233
Operative time, minute, mean (±SEM)	133.07 (±65.41)	218.75 (±105.74)	<0.001
PTC period, minute, mean (±SEM)	17.11 (±12.12)	29.25 (±13.95)	<0.001
Transfusion requirement, n (%)ESFFP	32 (16.9)128 (67.7)	40 (58.8)60 (88.2)	<0.0010.003
Reoperation, n (%)For bleedingFor other causes	1 (0.5)5 (2.6)	0 2 (2.9)	1.0001.000
Morbidity, n (%)AtelectasisPleural effusionPneumoniaPulmonary embolismPulmonary edemaSurgical site infectionDeep venous thrombosisLiver failureBiliary leakCardiac complications Ischemic heart attackArrhythmia	53 (28)40 (21.2)26 (13.7)8 (4.2)3 (1.5)6 (3.1)9 (4.7)1 (0.5)7 (3.7)6 (3.1)1 (0.5)0	29 (42.6)23 (33.8)15 (22.1)7 (10.2)2 (2.9)4 (4.8)6 (8.8)07 (10.2)11 (16.1)1 (1.5)1 (1.5)	0.0090.0060.1240.0770.6100.4630.2341.0000.0580.0010.172
Intensive care unit requirement	17 (8.9)	14 (20.5)	0.080
Postoperative hospital stay, days, median (min–max)	10 (2–97)	13 (3–75)	0.002
30-day mortality, n (%)	5 (2.6)	6 (8.8)	0.041
Follow-up, months, median (min–max)	35 (0–143)	31 (0–129)	0.089

## 4. Discussion

The critical threshold of bleeding amount that causes morbidity and mortality in liver surgery is unclear. Most trials have found that the average amount of blood loss in patients who undergo LR to is around 300 mL [14,15]. Our ROC curve analysis revealed that the cutoff point of operative bleeding was 500 mL. The cutoff point of bleeding during LR for disease treatment is completely different from that for donation. Ibrahim claimed that the morbidity of patients significantly increases with a bleeding amount of as low as 170 ± 79 mL during donor hepatectomy [16]. However, Yang determined 800 mL as a cutoff point of the significant bleeding amount that leads to morbidity [17]. The featured points of previous studies were similar major LR rates, completely normal liver parenchyma in the first study, and the presence of an underlying liver disease with HCC in the second one. In another study, the morbidity rate significantly increases with a bleeding amount of more than 1000 mL during LR [18]. The patient spectrum in our study seemed similar, but the major LR rates were lower than those in a previous study. However, the study interval between the two series was completely different. The implementation of technological devices in surgical techniques has altered the amount of bleeding in liver surgery, especially in the 2000s compared with the 1990s. Therefore, the bleeding threshold for the determination of morbidity in liver surgery should be lower than 1000 mL in current studies. If the cutoff point of the bleeding amount would be optimized, then 200–800 mL should be set in liver resection for disease treatment.

Systematic metaanalyses have shown that low-CVP surgery reduces the amount of blood loss and the need for transfusion during a LR [14,15,19]. Operating time and hospital stay are likely shortened in resections performed under a low CVP [14]. Therefore, its practice is supported. However, some controversies regarding the optimum anesthetic technique for decreasing CVP have not been resolved. The stabilization of hemodynamic alterations in the case of profuse bleeding under a low CVP is the most difficult part of this method for LR. Intermittent PTC is developed to minimize the adverse effects of continuous PTC [6,20]. Intermittent PTC also permits a significant increase (almost doubling) in ischemia times that can be achieved with continuous PTC. However, repeated clamp removal during intermittent PTC may result in fluctuations of systemic blood pressure, multiple episodes of hepatic IR injury, and repeated bleeding from transection surfaces. A systematic metaanalysis has demonstrated that intermittent PTC reduces blood loss and transfusion requirement during LR compared with those of the controls. Although blood loss recorded between the methods of vascular occlusion does not differ, the application of intermittent PTC likely shortens the operating time compared with that of continuous PTC [14]. 

Numerous devices have been developed to improve exposure ability and replace clamp crushing [4]. In terms of blood loss, biliary leak and transection time, several small randomized trials have not clearly demonstrated the superiority of any technique [14]. Therefore, the clamp crushing remains the preferred technique for liver surgery because of its simplicity and reliability. Bipolar electrothermal vessel sealers are attractive devices for the ligation and division of vessels and biliary channels. Metaanalysis has also suggested the usefulness of this device in terms of decreasing in blood loss, biliary leak, and hospital stay compared with that of ligation with clip and ties [21]. Other advanced techniques for ligation are radiofrequency (RF)-dissecting sealer and stapler transection. However, RF-dissecting sealers cause high infection rates and bleeding complications, while stapler transection is costly and has a high rate of biliary leaks [4,22]. Topical hemostatic agents can be classified as hemostatic matrix agents (collagen, cellulose, gelatin, or microporous polysaccharide spheres), coagulation factor-based agents (fibrin sealant or topical thrombin), and combination agents [4]. Various interventions have also been reported in the literature, but a large heterogeneity of results indicates weak conclusions on the field [14,23]. Limited evidence has indicated that the application of combination agents is more efficacious than matrix agents alone [4]. 

Despite our intensive efforts devoted to preventing pulmonary complications, the rate of pulmonary complications in our practice increased from 20.8% to 27.2% during 6-year period [2]. However, the fatalities of PE and pneumonia in this period decreased from 100% to 60% and from 50% to 26.7%, respectively. The overall biliary leak rate of this study seems comparable with that of relevant randomized controlled trials in the field. The overall rate of PHLF seems comparable with that in the literature. However, our treatment policy was successful and had a high survival rate after PHLF. Aggressive attempts to resolving vascular problems related to surgery and the judicious use of nonbiological liver support in the treatment algorithm against liver failure are key points to explain the favorable recovery rates associated with PHLF [13,24].

The increasing bleeding amount during liver surgery is strictly correlated with major resection, long operative time, and prolonged PTC period in this study. The adverse effects of operative bleeding during liver surgery on morbidity and mortality were clearly demonstrated. The small sample size, the absence of randomization, and the low major LR rate were the main limitations of the study. 

In conclusion, different methods are used in combination to control bleeding during liver surgery. Each step of bleeding control in this study is based on well-accepted applications and trusted devices or topical agents. Morbidity and mortality rates and bleeding amounts are found within acceptable ranges indicated in the literature. Although intensive efforts have been devoted to controlling bleeding, operative bleeding remains a major determinant of morbidity and mortality in liver surgery. However, favorable outcomes obtained with diligent postoperative care can encourage surgeons to achieve better results. 

## Funding

This study was performed without any support from funding agencies in the public, commercial, or not-for-profit sectors.

## Authors’ contributions

M. Selim Bodur, Kadir Tomas, and Hakan Küçükaslan are responsible for data collection and patients’ follow-up. Serdar Topaloğlu and Adnan Çalık are liver surgeons of the study. Şükrü Oğuz is an interventional radiologist of the surgical team. Davut Dohman is an anesthesiologist of the surgical team. Erdem Karabulut is a biostatistician.

## Ethical approval

This study was approved by the institutional committee on human subjects (Date: December 10, 2018; Number: 268/2018). 

## References

[ref1] (2002). Improvement in perioperative outcome after hepatic resection: analysis of 1803 consecutive cases over the past decade. Annals of Surgery.

[ref2] (2013). Intensive pulmonary care after liver surgery: a retrospective survey from a single center. Transplantation Proceedings.

[ref3] (2015). The impact of perioperative red blood cell transfusions on long-term outcomes after hepatectomy for colorectal liver metastases. Annals of Surgical Oncology.

[ref4] (2016). Hemostasis and hepatic surgery. Surgical Clinics of North America.

[ref5] (2009). Pharmacological interventions to decrease blood loss and blood transfusion requirements for liver resection. Cochrane Database of Systematic Reviews.

[ref6] (2005). Vascular control during hepatectomy: review of methods and results. World Journal of Surgery.

[ref7] (2009). The vessel sealing system (LigaSure) in hepatic resection: a randomized controlled trial. Annals of Surgery.

[ref8] (2010). Combined blunt-clamp dissection and LigaSure ligation for hepatic parenchyma dissection: Postcoagulation technique. Journal of the American College of Surgeons.

[ref9] (2013). Efficacy and safety of hepatectomy performed with intermittent portal triad clamping with low central venous pressure. BioMed Research International.

[ref10] (2017). Incidence of and risk factors for incisional hernia after liver surgery performed with a J-shaped right subcostal incision. The American Surgeon.

[ref11] (2020). Is it rational to perform liver resection for patients with intermediate and advanced stages of hepatocellular carcinoma?. The American Surgeon.

[ref12] (2011). Posthepatectomy liver failure: a definition and grading by the International Study Group of Liver Surgery (ISGLS). Surgery.

[ref13] (2010). The current status of non-biologic liver support in the treatment of liver failure. Yoğun Bakım Dergisi.

[ref14] (2016). Methods to decrease blood loss during liver resection: a network meta-analysis. Cochrane Database of Systematic Reviews.

[ref15] (2014). Minimizing blood loss during hepatectomy: a literature review. Journal of Surgical Oncology.

[ref16] (2006). Intraoperative blood loss is a risk factor for complications in donors after living donor hepatectomy. Liver Transplantation.

[ref17] (2011). Risk factors influencing postoperative outcomes of major hepatic resection of hepatocellular carcinoma for patients with underlying liver diseases. World Journal of Surgery.

[ref18] (2004). Improving perioperative outcome expands the role of hepatectomy in management of benign and malignant hepatobiliary diseases. Annals of Surgery.

[ref19] (2015). Central venous pressure and liver resection: a systematic review and meta-analysis. The Official Journal of International Hepato-Pancreato-Biliary Association.

[ref20] (2012). Vascular occlusion or not during liver resection: the continuing story. Digestive Surgery.

[ref21] (2013). Technology-assisted versus clamp-crush liver resection: a systematic review and meta-analysis. Surgical Innovation.

[ref22] (2014). Radiofrequency-assisted versus clamp-crush liver resection: a systematic review and meta-analysis. Journal of Surgical Research.

[ref23] (2016). An update on topical haemostatic agents in liver surgery: systematic review and meta-analysis. Journal of Hepato-Biliary-Pancreatic Sciences.

[ref24] (2017). Enhancing hepatic microcirculation in postoperative hepatic failure with intra-arterial recombinant tissue plasminogen activator treatment. Experimental and Clinical Transplantation.

